# Personalized Use of Disease-Modifying Therapies in Multiple Sclerosis

**DOI:** 10.3390/pharmaceutics16010120

**Published:** 2024-01-17

**Authors:** Chi-Yan Lee, Koon-Ho Chan

**Affiliations:** 1 Department of Medicine, School of Clinical Medicine, LKS Faculty of Medicine, The University of Hong Kong, Queen Mary Hospital, 405B, 4/F, Professorial Block, 102 Pokfulam Road, Hong Kong; 2 Neuroimmunology and Neuroinflammation Research Laboratory, LKS Faculty of Medicine, The University of Hong Kong, Hong Kong; 3 Research Center of Heart, Brain, Hormone and Healthy Aging, LKS Faculty of Medicine, The University of Hong Kong, Hong Kong

**Keywords:** relapsing multiple sclerosis, disease-modifying therapies, efficacy, adverse effects, personalization, shared decision making

## Abstract

Multiple sclerosis is an important neurological disease affecting millions of young patients globally. It is encouraging that more than ten disease-modifying drugs became available for use in the past two decades. These disease-modifying therapies (DMTs) have different levels of efficacy, routes of administration, adverse effect profiles and concerns for pregnancy. Much knowledge and caution are needed for their appropriate use in MS patients who are heterogeneous in clinical features and severity, lesion load on magnetic resonance imaging and response to DMT. We aim for an updated review of the concept of personalization in the use of DMT for relapsing MS patients. Shared decision making with consideration for the preference and expectation of patients who understand the potential efficacy/benefits and risks of DMT is advocated.

## 1. Introduction

Multiple sclerosis (MS) is a central nervous system (CNS) inflammatory demyelinating disorder which can lead to severe and permanent neurological disabilities, especially in young patients. The majority of patients (~85%) have a relapsing–remitting course at clinical onset of the disease which is characterized by recurring acute attacks of inflammation affecting various regions of the CNS. These acute attacks of inflammatory demyelination are accompanied by axonal injury even in the early stage of the disease, with clinical recovery associated with partial remyelination and axonal regeneration [1]. Acute attacks of inflammation with or without clinical relapses are more prominent in the early stage of relapsing–remitting multiple sclerosis (RRMS) and occur in areas of demyelination, typically around post-capillary venules characterized by the breakdown of the blood–brain barrier (BBB). The breach of the BBB increases the trans-endothelial migration of activated leucocytes including T and B lymphocytes and macrophages into the CNS, leading to further inflammation and demyelination followed by oligodendrocyte loss, reactive gliosis, and neuro-axonal degeneration [2,3]. Recent evidence suggests that progression independent of relapse activity (PIRA) is a substantial contributor to long-term disability accumulation in RRMS [4]. A recent systematic review involving 48 eligible studies revealed that PIRA was reported to occur in about 5% of RRMS patients per annum, causing at least 50% of all disability accrual events in typical RRMS. The proportion of PIRA vs. relapse-associated worsening increased with age, longer disease duration, and, despite lower absolute event numbers, the potent suppression of relapses by a high-efficacy DMT. The authors conclude that PIRA is the most frequent manifestation of disability accumulation across the full spectrum of traditional MS phenotypes, including clinically isolated syndrome (CIS) and early RRMS [5]. 

In the later stage of MS, acute inflammation becomes less frequent and prominent, whereas axonal injury/loss and neuronal loss accumulate from the degeneration of chronic demyelinated axons secondary to energy deficit from mitochondrial dysfunction and the loss of myelin trophic support [1]. When RRMS patients develop irreversible and progressive deterioration of neurological functions with or without clinical relapses in the later stage of the disease, traditionally described as conversion to secondary progressive multiple sclerosis (SPMS) [1], the contribution of the peripheral immune system decreases, and the immune response is characterized by CNS-compartmentalized inflammation involving CD8+ T cells and plasma cells that survive and persist in the CNS and surrounding meninges, an microglial and astrocytic inflammatory response [6,7]. CNS pathologies changes from focal to diffuse white matter injury with microglial activation, diffuse lymphocytic and monocytic infiltrates, and increasing cortical involvement thought to be associated with lymphoid-like follicles (ectopic germinal centers) in the meninges [8,9]. 

## 2. Etiology, Risk Factors, and Prognostic Factors

The exact etiology of MS is uncertain. Genetic factors affect susceptibility to MS, and environmental factors interact with genetic factors [10]. Genomic-wide association studies identified >200 genetic risk variants for MS; each has a small effect on risk of disease, and most of these variant encode molecules involved in the immune system focusing on T cells, B cells, and microglia [11,12,13,14]. Lifestyle and environmental factors are important risk factors for MS, and substantial evidence supports a period of susceptibility to environmental risk factors for MS during adolescence [15]. These include Epstein–Barr virus (EBV) infection in adolescence and early adulthood, tobacco exposure via active or passive smoking, a lack of sunlight exposure, low vitamin D levels, teenage obesity, and other less well-established risk factors including night work and excessive alcohol or caffeine consumption [16]. Most recently, strong evidence supports that EBV infection is a needed but insufficient risk factor for the development of MS [17,18]. An elegant study demonstrated high-affinity molecular mimicry between the EBV transcription factor EBV nuclear antigen 1 (EBNA1) and the CNS protein glial cell adhesion molecule (GlialCAM). A cross-reactive CSF-derived antibody was identified by single-cell sequencing the paired-chain B cell repertoire in the blood and CSF of MS patients. Subsequent protein microarray-based testing of recombinantly expressed CSF-derived antibodies against MS-associated viruses, a sequence analysis, affinity measurements, and the crystal structure of the EBNA1-peptide epitope in complex with the autoreactive Fab fragment enabled tracking of the development of the naive EBNA1-restricted antibody to a mature EBNA1-GlialCAM cross-reactive antibody. Anti-EBNA1 and anti-GlialCAM antibodies are prevalent in patients with MS. The results of this study provide a mechanistic link for the association between MS and EBV [19].

Importantly, MS patients are heterogeneous in pathogenetic mechanisms, CNS pathologies, immunopathobiologies, clinical features and severity, response to immunotherapies, and prognosis [2,10,20,21]. A number of epidemiological, lifestyle and environmental factors, clinical, neuroradiological, and laboratory characteristics have been identified as good (female sex, younger onset age, and onset with optic neuritis or somatosensory dysfunction) or poor prognostic factors for disability progression in MS. Table 1 summarizes the poor prognostic factors for relapsing MS. 

## 3. Roles of T Cells, B Cells, and Myeloid Cells in Multiple Sclerosis Pathophysiologies 

### 3.1. T Lymphocytes

MS was considered a predominantly T cell-mediated CNS IDD for years until recently, when B cells were also recognized to be important. T cell abnormalities detected in MS include an increased number of circulating CD4+ Th1 and Th17 cells with enhanced effector functions [22,23], insufficient function of regulatory T cells, decreased expression of forkhead box protein P3 (FOXP3) by regulatory T cells [24], and the resistance of CNS-specific effector T cells to regulatory T cell-mediated regulation [25]. CD8+ T cells also play a role, especially in the compartmentalized inflammation described in SPMS [26,27]. A recent study on pediatric MS focusing on T cell subsets confirmed deficient regulatory T cell functions, increased proinflammatory T cell responses, and the resistance of effector T cells to regulatory T cell suppression in MS [28].

### 3.2. B Lymphocytes

The presence of oligoclonal bands (OCBs) detected in the CSF but not in the serum of MS patients has raised consideration of the role of B cells in MS for several decades. As T cells play important roles in MS pathophysiologies and B cells are required for the activation and development of T cells via antigen presentation and proinflammatory cytokine secretion, it is expected that B cells play an important role in MS pathophysiology. This is strongly supported by the effectiveness of B cell depletion via CD20 monoclonal antibodies (rituximab used off-label and then proven for ocrelizumab in RCT) [29,30]. B cell abnormalities reported in MS patients include an abnormal propensity to produce proinflammatory cytokines including IL-6, GM-CSF, TNF, and lymphotoxin-α and a deficiency in regulatory cytokines such as IL-10 [31,32,33]. It is believed that in MS, B cells contribute to the early activation of peripheral T cells and the activation of T cells and myeloid cells in the CNS through antigen presentation and the secretion of proinflammatory cytokines and chemokines [16,20]. A recent study demonstrated that peripheral memory B cells are critical for the increased autoproliferation of T cells (spontaneous proliferation in the absence of exogenous stimulus) observed in MS patients and the homing of activated and autoreactive T cells to migrate to the CNS via crossing the BBB and mediate acute neuroinflammation with/without clinical relapse [34]. This abnormally increased autoproliferation of T cells is partly driven by human leucocyte antigen-dependent interactions with memory B cells, which are more efficient APCs, and is reduced after anti-CD20 monoclonal antibody treatment [12]. B cells are increased in the peripheral blood of MS patients compared with matched controls, and these cells are found in their CSF, meninges, and blood [35,36].

### 3.3. Myeloid Cells 

Circulating macrophages and CNS-resident microglia are important cells of the innate immune system. Both are APCs with the capacity to secrete growth factors and inflammatory mediators (cytokines, chemokines, and ROS); hence, they can activate T cells. Microglia are resident phagocytes of the CNS and play important roles in mammalian CNS development, the maintenance of neuronal function via neuron–glia communication, the clearance of toxic proteins and cellular debris, defense against invading microbes, and response to trauma. In autoimmune and neurodegenerative diseases, reactive microglia contribute to disease pathologies and progression via the secretion of inflammatory mediators such as cytokines, chemokines, and reactive oxygen species. Microglia have diverse activation states in MS and can be both detrimental and beneficial [37,38]. The microglial inflammatory response likely contributes to CNS inflammation and neuronal injury/cytotoxicity in MS [39] and is a potential target for therapeutic development [40].

Importantly, there is substantial biological heterogeneity across MS patients. Different patients likely harbor different and variable degrees of abnormalities in the cellular responses of T cells, B cells, and myeloid cells, and the summation effect of their interactions results in disease development and possibly periods of enhanced disease activity [20]. This immune biological heterogeneity likely explains the diverse variability in clinical features, response to DMT, and prognosis of MS patients [10] and is probably related to underlying genetic heterogeneity and the complex interactions between genetic and environmental factors.

## 4. Disease-Modifying Therapies

MS patients and their caring neurologists have been excited in the past two decades as more than ten disease-modifying drugs have become available since beta-interferon 1b was introduced as a DMT for RRMS in 1993. DMTs aim to reduce clinical relapse frequency, slow the accumulation of neurological disabilities, and prevent the development of SPMS in relapsing MS patients, presumably via the prevention of acute inflammatory attacks on the CNS and the suppression of CNS inflammation. Increasingly, potent DMTs have been developed and approved by the Food and Drug Administration (US) and European Medicine Agency (Europe); these include oral drugs (teriflunomide, dimethyl fumarate, fingolimod, siponimod, ozanimod, and cladribine) that relieve patients from discomfort/adverse effects associated with subcutaneous injection and the intravenous infusion of monoclonal antibodies (natalizumab, alemtuzumab, rituximab, and ocrelizumab) that are helpful for patients who have suboptimal compliance with frequent subcutaneous injections or daily oral medications.

Importantly, DMTs for MS can be classified according to effectiveness into low-efficacy, moderate-efficacy, moderate- to high-efficacy, and high-efficacy groups [16,41,42,43] (Table 2). In addition, they vary in pharmacological mechanisms and can conceptually be broadly classified into either continuous medications (beta-interferon, glatiramer acetate, teriflunomide, dimethyl fumarate, sphingosine-1-phosphate modulators, natalizumab, rituximab, and ocrelizumab) with effects which subside upon withdrawal and pulsed therapies followed by immune reconstitution (alemtuzumab and cladribine) with long-term changes in lymphocyte repertoire that may confer transient remission of the disease (Table 3).

### 4.1. Low-Efficacy Disease-Modifying Therapies

#### 4.1.1. Interferon Beta

Interferon beta (IFNβ), consisting of IFNβ1a (subcutaneously three times per week or intramuscularly weekly) and IFNβ1b (subcutaneously on alternate days), are the first approved DMTs for RRMS. These injectables require frequent injection, and the frequency is reduced with a pegylated formulation to once every 2 weeks [44]. The therapeutic effects are related to the downregulation of MHC class II expression on APC, the induction of IL-10 production by T cells leading to a shift in T helper (Th) cell response to the anti-inflammatory Th2, and the inhibition of T cell migration via the blockade of metalloproteinases and adhesion molecules [45]. Double-blinded, placebo-controlled trials have confirmed their modest efficacy of reducing ARR by 31–34%, slowing disability progression and preventing SPMS. In a 16-year follow-up study of the initial trial for IFNβ1b, mortality rates were significantly lower in the IFNβ group (5.6% for the 8 MIU group, 9.3% for 1.6 MIU group, and 18.3% for the placebo group). Out of 113 patients that reached an EDSS of 6.0, 45.6% were originally assigned to the placebo, 38.8% were assigned to IFNβ1b 1.6 MIU, and 45.8% were assigned to IFNβ1b 8 MIU. This analysis did not consider death part of disability outcome, skewing results against the treatment arm [46,47]. Similarly, in a 21-year follow-up analysis, IFNβ1b treatment was associated with a 46.8% reduction in long-term all-cause mortality compared to a placebo [48]. An RCT of IFNβ1a administered subcutaneously three times per week showed that low-dose (22 ug) and high-dose (44 ug) treatment reduced the ARR by 27% and 33%, sustained disability progression by 22% and 30%, and reduced the accumulation of T2 active lesions on MRI by 67% and 78%, respectively, compared to a placebo [49]. A subsequent extension study confirmed the superiority of the high dose in reducing clinical relapse and the prevention of disability and supported the importance of early treatment initiation [50]. Interestingly, 382 patients of the original 560 enrolled patients continued into the long-term, open-label follow-up study which showed that the high-dose group required the longest time to reach a confirmed EDSS of 4.0 and a reduction in brain volume loss (BVL) over the 8 years compared to both the low-dose and placebo groups [51]. A further post hoc analysis highlighted long-term compliance as an important outcome factor as patients receiving the maximum dose and with maximum adherence had lower ARRs, lower SPMS conversion rates, and lower T2 lesion loads [52]. Pegylated IFNβ1a once every 2 weeks conferred a similar reduction in the ARR, disease activity on MRI (gadolinium-enhancing lesions, −0.26 vs. 0.77; new T2 lesions, 3.7 vs. 11.2; T1 hypointense lesions, 1.8 vs. 3.8), and sustained disability progression (HR 0.62) compared to a placebo [44]; an extension study further revealed the treatment’s efficacy as patients originally assigned to the treatment arm outperformed those who switched after year 1 [53]. NEDA-3 was achieved in 33.9% of pegylated IFNβ1a once every 2 weeks versus 15.1% for a placebo [54].

Their long-term safety is confirmed without the risk of significant infection, including progressive multifocal leukoencephalopathy (PML). Despite common adverse effects including liver function derangement [55], injection site reactions, flu-like symptoms, myalgia, and depression, IFNβs are usually well tolerated [56] and remain an option for RRMS and relapsing SPMS with mild disease activity and severity. IFNβ1a and 1b are non-teratogenic and can be continued in relapsing MS patients until a positive pregnancy test and during the first trimester [57]; if the disease is more active, they can be continued during pregnancy [58,59]. Response to vaccines is not affected by IFNβ, and live attenuated vaccines are not contraindicated in patients on regular IFNβ therapy. In addition, post-marketing real world data show no increased risk of malignancy with long-term therapy [60]. During the recent COVID-19 pandemic, IFNβwas additionally favored for its anti-coronavirus effect.

#### 4.1.2. Glatiramer Acetate

Glatiramer acetate (GA) is a synthetic copolymer composed of four amino acids found in myelin basic protein [61]. Although the exact mechanism of action is not completely understood, it is known to involve both innate and adaptive immunity, exerting immunomodulatory effects on T cells, B cells, monocytes, and various cytokine pathways [61].

Initially, the FDA approved glatiramer acetate for RRMS in 1996. The indications were later extended to include clinically isolated syndrome (CIS) and active SPMS in adults. The medication is administered via subcutaneous injection, either at a dose of 20 mg daily or 40 mg three times a week.

In a multicenter phase III double-blind, placebo-controlled trial involving 251 patients with RRMS, glatiramer acetate reduced the annualized relapse rate by 29% [62]. Another randomized placebo-controlled trial with 239 RRMS patients undergoing monthly MRI assessments found a 35% reduction in the total number of enhancing lesions and a 33% reduction in the relapse rate with glatiramer acetate treatment [63]. The more recent GALA study evaluated the efficacy and safety of glatiramer acetate administered three times per week and reported a 34% reduction in relapses, a 44.8% reduction in gadolinium-enhancing T1 lesions, and a 34.7% reduction in new or newly enlarging T2 lesions [64]. The PreCISe trial, which studied patients with CIS, demonstrated that glatiramer acetate treatment reduced the risk of developing clinically definite MS by 45% compared to a placebo [65]. Additionally, the 5-year open-label phase of the PreCISe trial indicated that early treatment with glatiramer acetate was associated with a longer delay in conversion to clinically definite MS, less brain atrophy, fewer new T2 lesions per year, and a lower T2 lesion volume [66].

Glatiramer acetate’s adverse effects include injection-site reactions and immediate post-injection reactions such as flushing, chest tightness, dyspnea, palpitations, tachycardia, urticaria, and anxiety. These post-injection reactions are typically self-limited and require no treatment. Rarely, lipoatrophy at injection sites can occur after prolonged use, which may necessitate treatment cessation. Like IFNβ, glatiramer acetate is not considered immunosuppressive and is not associated with an increased risk of opportunistic infections, PML, or malignancy. It is safe for use during pregnancy [59] and is not associated with significant laboratory abnormalities; hence, regular monitoring is not required. Glatiramer acetate is deemed safe for women during conception, throughout pregnancy, and while breastfeeding, presenting no elevated risk of birth defects or fetal loss. A database of exposure to branded glatiramer acetate in more than 7000 pregnancies in women with MS revealed no increased risk of congenital anomalies in comparison to the general population [67].

#### 4.1.3. Teriflunomide

Teriflunomide (TF) is an oral inhibitor of dihydroorotase dehydrogenase, a mitochondrial enzyme necessary for de novo pyrimidine synthesis, which is required for the expansion of antigen-activated lymphocytes. Through the inhibition of pyrimidine synthesis, it leads to reduced activity of proliferating T and B cells, a lower Th1 cell count, and an increased CD4+/CD8+ T cell ratio and regulatory T cell count [68]. Randomized controlled trials (TEMSO and TOWER studies) show that teriflunomide reduced the ARR by 31% and 36%, respectively, disease activity on MRI (reducing the total lesion volume by 67.4% in TEMSO), and the proportion of patients with sustained disability progression (20.2% vs. 27.3% in TEMSO and an HR of 0.68 in TOWER) compared to a placebo in RRMS patients [69,70]. Teriflunomide has been compared to other DMTs in phase III studies. The TENERE study and its extension, comparing teriflunomide to IFNβ1a, reported significant improvement in patient satisfaction related to adverse effects and convenience after switching from IFNβ1a to teriflunomide [71]. The OPTIMUM study compared ponesimod to teriflunomide in relapsing MS patients; at week 108, the ponesimod group had a lower ARR compared to the teriflunomide group (0.202 vs. 0.290) and improved fatigue scores [72]. In ASCLEPIOS I and II, the ofatumumab group had a lower ARR, disease activity on MRI, and worsening rates of disability but similar rates of clinical disability improvement and brain volume loss (BVL) reduction compared to the teriflunomide group [73]. Teriflunomide has a robust beneficial effect on neurodegeneration evidenced by the preservation of brain volume and a reduction in disability progression [74,75]. In the TOPIC study, 2-year treatment with teriflunomide resulted in lower rates of cortical-gray-matter and whole-brain atrophy compared to a placebo, and every 1% reduction in brain volume was associated with a corresponding 14.5% (gray matter) and 47.3% (whole brain) increased risk of conversion from CIS to clinically definite MS [75].

The drug is convenient for its once-daily dose and bears no risk for PML. It is shown to be teratogenic in animal studies (FDA), and prescription guidelines require effective contraception in both female and male patients. Common adverse effects include hair loss, headache, nausea, diarrhea, nasopharyngitis, urinary tract infection, and elevated ALT, whereas rare but potentially severe adverse effects include hepatotoxicity, opportunistic infections (aspergillosis), bone marrow suppression, elevated blood pressure, peripheral neuropathy, and interstitial lung disease [76]. Before the initiation of therapy, the patient should be vaccinated as needed and screened for latent tuberculosis infection, especially in regions endemic for tuberculosis. Liver function tests must be conducted monthly for 6 months after initiation [77,78]. 

### 4.2. Moderate-Efficacy Disease-Modifying Therapies

#### Dimethyl Fumarate

Dimethyl fumarate (DMF) is the methyl ester of fumaric acid. Its exact mechanism of action in MS is not fully understood, but it is believed to exert its effects through a combination of anti-inflammatory and neuroprotective mechanisms. DMF and its active metabolite, monomethyl fumarate (MMF), activate the nuclear factor (erythroid-derived 2)-like 2 (Nrf2) pathway, which plays a key role in the cellular response to oxidative stress [79]. This can lead to reductions in inflammation and oxidative damage to the nervous system, both key factors in the pathogenesis of MS. In 2013, DMF was approved by the FDA for CIS, RRMS, and active SPMS in adults. The standard regimen is 240 mg taken orally twice daily.

The DEFINE trial was a pivotal randomized, double-blind, placebo-controlled phase III trial that evaluated the efficacy and safety of DMF in RRMS. It involved 1234 patients who were randomized to receive either 240 mg of DMF twice daily, 240 mg of DMF three times daily, or a placebo. Both DMF regimens were shown to reduce the ARR by 53% (twice daily) and 48% (thrice daily), respectively [80]. The rate of disability progression and the number of lesions were also reduced compared to the placebo group. The CONFIRM trial had a similar study design to the DEFINE trial but included glatiramer acetate as a comparator treatment arm. The ARR was significantly lower compared to the placebo group, with relative risk reductions of 44% for twice-daily DMF, 51% for thrice-daily DMF, and 29% for glatiramer acetate [81]. The numbers of new or enlarging T2-weighted hyperintense lesions and new T1-weighted hypointense lesions were also significantly reduced in all three active treatment arms.

The adverse effects of DMF include flushing, hot flashes, and gastrointestinal disturbances. The flushing can be mitigated by taking the medication with food or using aspirin in severe cases. Gastrointestinal disturbances typically improve over time, and treatment is mainly symptomatic (e.g., loperamide for diarrhea). Other adverse effects include hepatotoxicity, lymphopenia, and infections, including herpes infections and rare cases of PML. PML was associated with prolonged severe lymphopenia and older age [82]. The FDA’s DMF label includes a recommendation to consider treatment interruption in patients with absolute lymphocyte counts of less than 0.5 × 10^9^/L persisting for ≥6 months. Although other opportunistic infections have been reported, a long-term safety follow-up study showed that the incidence of serious infection in patients with prolonged severe lymphopenia was not higher than in patients with normal lymphocyte counts [83]. Periodic monitoring of complete blood counts (CBCs) with a differential as well as liver function tests are recommended during DMF therapy at least every 6 months. Although there is no report of potential risks or fetotoxicities in pregnancy associated with DMF, the available evidence is too scarce for any conclusions; it is not recommended during pregnancy and should be avoided unless it is clearly necessary and the potential benefits outweigh the risk to the fetus [84].

Diroximal fumarate is an oral bioequivalent to DMF with reduced gastrointestinal disturbances. Monomethyl fumarate is the major active metabolite of DMF with improved gastrointestinal tolerability. Both are approved by the FDA for CIS, RRMS, and active SPMS. 

### 4.3. Moderate- to High-Efficacy Disease-Modifying Therapies

#### 4.3.1. Sphingosine-1-Phosphate Receptor Modulators

Sphingosine-1-phosphate (S1P) binds to S1P receptors on the surface of lymphocytes, and the subsequent signaling activity is important for lymphocyte egress from secondary lymphoid organs. S1P receptor modulators including fingolimod, siponimod, and ozanimod bind to S1P receptors and lead to the internalization and degradation of S1P receptors. These result in the trapping of lymphocytes in the secondary lymphoid tissues and hence peripheral lymphopenia [84].

##### Fingolimod

There are five types of S1P receptors (S1PR 1-5, and S1PR1 is abundantly expressed in lymphocytes), and fingolimod binds to four of the five types with high affinity to S1PR1. The FREEDOMS I and II trials demonstrated the clinical efficacy of fingolimod in reducing the ARR by 54%, reducing the development of new or enlarging lesions on MRI by 74%, and slowing confirmed disability progression and BVL compared to a placebo at 2 years [85,86]. Fingolimod was shown to be superior to weekly interferon-β1a in reducing the ARR in relapsing MS in the TRANSFORMS trial [87]. Patients should be checked for antibodies against varicella zoster virus (VZV) before initiation, and seronegative patients should be vaccinated before initiation. Fingolimod is taken once orally at 0.5 mg, and live attenuated vaccines should be avoided in patients on fingolimod therapy. Patients need to be screened for bradycardia, long QT, AV block, and other arrhythmia risk factors before initiation, the first dose must be given as an in-patient treatment for hourly blood pressure measurements, and cardiac monitoring for bradycardia from atrioventricular block (related to fingolimod binding to S1PR3) can occur. 

Macular edema is a rare complication which requires regular ophthalmological assessments for all patients taking fingolimod. The peripheral lymphocyte count and liver function should be monitored regularly. Severe lymphopenia (<200/µL) and prolonged severe lymphopenia (<500/µL) require drug withdrawal or a dose reduction as rare cases of PML have been reported to be related to fingolimod therapy. The risk of PML is about~1:12,000 and increases with older age and a longer duration of therapy [88], and the risks of other opportunistic infections, including herpes virus and cryptococcus infection, are increased. Long-term therapy with fingolimod slightly increases the risk of malignancy, predominantly basal cell carcinoma of the skin, and patients with active malignancy should avoid its use. Fingolimod is considered a DMT of moderate-to-high efficacy. It is approved in the US as a first-line DMT for relapsing MS and as a second-line DMT in Europe.

##### Siponimod

Siponimod is a newer S1P receptor modulator which binds to S1PR5; hence, it is less likely to induce cardiac conduction abnormalities. Its half-life (7 h) is shorter than that of fingolimod, requiring a shorter duration of washout (6–9 days) when switching to other DMT. Siponimod is approved for RRMS and SPMS with active disease based on results of the BOLD (a phase 2 study with 188 RRMS patients enrolled) and EXPAND trials [89,90]. In the EXPAND trial, 1651 SPMS patients with EDSS scores from 3.0 to 6.5 and no evidence of relapse in the 3 months before study initiation were randomized to siponimod or a placebo at a 2:1 ratio. The results showed that the siponimod group had a significant reduction in confirmed disability progression at 6 months (*p* = 0.0058), and a subgroup analysis identified younger age, a lower baseline EDSS score, shorter disease duration, and signs of active inflammation as factors associated with response to therapy [89]. Its metabolism is dependent on CYP2C9, and dosing requires genetic testing for CYP2C9 alleles; as the CYP2C9*3 variant is associated with reduced metabolism, heterozygotes for CYP2C9*3 should receive half of the maintenance dose, and homozygotes are contraindicated for siponimod. With gradual dose titration dose (0.25 mg/day on day 1, 0.5 mg day 3, 0.75 mg day 4, and 1.25 mg day 5 followed by a maintenance dose of 2 mg daily), first-dose observation monitoring is not required in patients without pre-existing cardiac disorders. Its long-term adverse effects including a higher risk of infection and malignancy are similar to fingolimod, though long-term post-marketing real world data are awaited.

##### Ozanimod

Ozanimod is an S1P receptor modulator for the treatment of RRMS. It binds to S1PR1 and S1PR5 and prevents the egress of lymphocytes from secondary lymphoid tissues [91]. The phase III RADIANCE part B study (24 months) and the SUNBEAM study (12 months) compared ozanimod to weekly IFNβ1a and showed that the ozanimod group had a lower ARR, disease activity on MRI, and a reduced rate of brain atrophy compared to the IFNβ1a group, but CDP results were similar [92,93]. Ozanimod has no cardiac adverse events, macular edema or serious infections reported in these clinical trials. Similar to siponimod, no first-dose observation is required for patients without pre-existing cardiac conditions when ozanimod is up-titrated (0.23 mg for days 1–4, 0.46 mg for days 5–7, and 0.92 mg once daily thereafter).

##### Ponesimod

Ponesimod is the most recently approved S1P receptor modulator for the treatment of RRMS. It has a high affinity for S1PR1 and a short half-life of 32 h. After discontinuation, the lymphocyte count will normalize within 7 days. Transient bradycardia and atrioventricular blocks occur in about 2%, and dyspnea and respiratory side effects may occur. It is approved by the FDA for CIS, RRMS, and active SPMS [94].

#### 4.3.2. Cladribine

Cladribine is an antimetabolite that specifically accumulates in both T and B lymphocytes, inhibits DNA synthesis and repair, and promotes cellular apoptosis without having a major impact on cells of the innate immune system [95]. Its cytotoxic effects on lymphocytes may reduce inflammation and relapse in MS. Cladribine was granted FDA approval in 2019 for adults with RRMS or active SPMS. The recommended regimen is 3.5 mg per kilogram body weight, administered orally. Usually, two treatment courses (each course being 1.75 mg/kg) are given yearly. Each treatment course is divided into two treatment cycles of four or five days, approximately four weeks apart. This dosing schedule allows for convenient oral administration with a relatively low number of dosing days. The efficacy of oral cladribine in MS was demonstrated in the CLARITY study, a phase III randomized, placebo-controlled clinical trial. Patients with RRMS were randomized to receive either 3.5 mg/kg of cladribine, 5.25 mg/kg of cladribine, or a placebo. The relative reductions in the annualized relapse rate were 57.6% and 54.5% for the 3.5 mg group and the 5.25 mg group, respectively, compared to the placebo [96]. Oral cladribine was also significantly associated with a higher relapse-free rate and reductions in the brain lesion count on MRI. In the extension study, placebo recipients from CLARITY received 3.5 mg/kg of cladribine, and cladribine recipients were re-randomized 2:1 to 3.5 mg/kg of cladribine or a placebo, with the blind maintained. It was shown that cladribine treatment for 2 years followed by 2 years of placebo treatment produced durable clinical benefits similar to 4 years of continuous cladribine treatment, with a low risk of severe lymphopenia or clinical worsening [97]. However, no obvious improvements were observed with further cladribine treatment after the initial 2-year treatment period in the study.

Common adverse effects include lymphopenia, upper respiratory tract infections, headache, and nausea. The lymphocyte count typically drops 2–3 weeks after cladribine therapy, reaches its nadir 2–3 months after treatment initiation, and then gradually recovers in the following months. Anti-herpes prophylaxis is recommended for those with lymphocyte counts less than 200/µL. The European Union Summary of Product Characteristics for cladribine tablets states that treatment course in the second year can be delayed for up to 6 months to allow for lymphocyte recovery (at least 0.8 × 10^9^/L). However, if recovery takes more than 6 months, the patient should not receive further treatment [98].

Before starting cladribine, patients should be screened for active or latent human immunodeficiency virus (HIV), tuberculosis, hepatitis B, hepatitis C, and other acute infections. Patients who are varicella zoster virus antibody-negative should be vaccinated before treatment. Cladribine is contraindicated for those with an active malignancy. Three cases of cancers were reported in the 3.5 mg/kg cladribine group in the CLARITY study compared to zero in the placebo group [96]. Although a subsequent meta-analysis did not support an increased cancer risk from cladribine in the doses used in the clinical trials [99], the potential increased risk of cancer should be discussed with patients. Oral cladribine is contraindicated for use during pregnancy and lactation. Effective contraception should be used while taking cladribine. As oral cladribine may reduce the effectiveness of oral contraceptives, a barrier contraceptive should be added during, and for at least 4 weeks after, each treatment course. Men with the potential for reproduction should also use effective contraception when taking oral cladribine. 

### 4.4. High-Efficacy Disease-Modifying Therapies

#### 4.4.1. Natalizumab

Natalizumab is an IgG4 humanized monoclonal antibody targeting the very late antigen-4 (VLA4) expressed on the surface of lymphocytes. VLA4 binds to vascular cell adhesion molecule 1 (VCAM1) expressed on endothelial cells and is critical for the migration of lymphocytes from peripheral circulation to the CNS. The AFFIRM trial showed that a monthly intravenous infusion of natalizumab (every 28 days) significantly reduced the ARR by 68% (0.26 versus 0.81), sustained disability progression by 54% and reduced gadolinium-enhancing lesions on MRI by >90% compared to a placebo at 2 years [100]. As an add-on therapy to IFNβ1a for RRMS patients with active disease while on IFNβ1a, natalizumab was shown to reduce the ARR by 54% and risk of sustained disability progression compared to a placebo at 2 years in the SENTINEL trial [101]. Common adverse effects of natalizumab include infusion reactions and infections such as respiratory tract and urinary tract infections [102,103]. Other immunomodulators should be stopped at least one month before the initiation of natalizumab. An anti-drug antibody is reported to develop in ~6% of patients and lead to poor treatment response to the drug [104].

Natalizumab is one of the most potent DMTs for relapsing MS and is generally considered to be a second-line or even third-line drug due to the risk of PML, a potentially life-threatening complication from the opportunistic infection of oligodendrocytes by the polyoma virus (John Cunningham virus (JCV)). The risk of PML associated with natalizumab therapy is in general 4.2 per 1000 depending on the duration of treatment, exposure to JCV (measured using the JCV antibody index; high if >0.9), and prior immunosuppressant therapy. However, natalizumab can be considered a first-line therapy for highly active MS patients aiming for rapid disease control, especially with the reported benefit of confirmed disability improvement (CDI) in a proportion of patients. Patients seronegative for the JCV antibody have negligible risk of PML (~1 in 10,000) and long-term therapy can be considered. For patients seropositive for the JCV antibody without prior immunosuppressant therapy, the risk of PML is estimated to be <1/1000 in the first 24 months of natalizumab therapy and increases to up to 4/1000 by 72 months of therapy (TYSABRI 2022), with further risk stratification according to the JCV antibody index [102].

The JCV antibody index should be checked regularly at 6-month intervals in patients on natalizumab therapy. Those who become seropositive should consider switching to another DMT, especially as treatment duration reaches 2 years, to avoid PML. The discontinuation of natalizumab has been reported to be associated with MS disease reactivation or rebound [105]. A washout period of 8 to 12 weeks is associated with a lower risk of disease reactivation or rebound compared to the initiation of another DMT after 16 weeks [106]. Recently, extended-dose natalizumab, such as a 300 mg intravenous infusion every 6–8 weeks, has been reported to be associated with a lower risk of PML [42,43]. Natalizumab-sztn (Tyruko), a biosimilar of natalizumab, was recently approved in August 2023 by the FDA for highly active RRMS, and its clinical benefits and side effects are same as that of natalizumab.

#### 4.4.2. Alemtuzumab

Alemtuzumab is a monoclonal antibody that targets the CD52 antigen, a protein found on the surface of mature lymphocytes but not on stem cells. Its mechanism of action in MS is tied to the depletion of circulating T and B lymphocytes, which are believed to instigate inflammation and relapses in MS. The rates of repopulation vary among CD52+ cell types, with monocytes returning to pretreatment levels within the first month and B cells fully recovering within 3 months and exceeding baseline numbers at 1 year. The recovery of T-cell subsets is slower, with CD8+ and CD4+ T cells taking about 12 months to reach the lower limits of normal [107,108].

Alemtuzumab received FDA approval in 2014 for adults with relapsing MS. It is administered via intravenous infusion at 12 mg daily for five consecutive days, followed by a second course of 12 mg daily for three days a year later. Subsequent treatment courses (12 mg daily for three days) are given as needed at least 12 months after the last dose. Premedication with 1 g of intravenous methylprednisolone daily for the first three days of each course is recommended.

Three pivotal trials, a phase II randomized, IFN-β-compared trial (CAMMS223) and two phase III randomized, IFN-β-compared trials (CARE-MS I and CARE-MS II), evaluated the efficacy and safety profile of alemtuzumab in relapsing MS [109,110,111]. These trials were single-blinded, with patients aware of their treatment group but assessed by blinded raters. Compared to 44 μg of interferon beta-1a subcutaneously three times a week, alemtuzumab reduced the annualized relapse rate from 49% to 55% in the two phase III trials. Alemtuzumab also reduced the sustained accumulation of disability by 42% in the CARE-MS II trial, which recruited patients who had previously relapsed despite their first-line treatment. In the five-year extension study, 59.8% received no alemtuzumab retreatment after the initial two courses, maintaining low ARR and BVL values [112].

Serious adverse effects include infusion reactions (occasionally severe and potentially fatal), infections, and the development of autoimmunity. Anti-herpes virus prophylaxis must be given from the first day of the treatment period until either two months after the completion of the treatment or until the CD4+ lymphocyte count is more than 200/µL, whichever occurs last. Other opportunistic and potentially fatal infections include listeriosis, toxoplasmosis, nocardiosis, aspergillosis, pneumocystis pneumonia, candidiasis, tuberculosis reactivation, and PML [113]. Vaccinations should take place two to four weeks before alemtuzumab treatment or at least six months afterward [114]. Live attenuated viral vaccines should not be given after a course of alemtuzumab until the lymphocyte counts recover. It is also recommended to screen patients for HIV, hepatitis B, hepatitis C, and tuberculosis and provide appropriate treatment as necessary prior to alemtuzumab treatment.

Secondary autoimmune conditions after treatment with alemtuzumab include frequent thyroid conditions (40%, hypothyroidism and hyperthyroidism) and rare but serious conditions such as immune thrombocytopenia (3.2%) and anti-glomerular basement membrane disease (0.2%) [113]. Other rare adverse events include stroke, as reported in post-marketing surveillance. The Risk Evaluation and Mitigation Strategy (REMS) program for alemtuzumab requires monthly monitoring of blood counts with differential, serum creatinine levels, and urinalysis with urine cell counts, as well as a thyroid test every three months until 48 months after the last dose. Due to its safety concerns and risk profile, alemtuzumab is generally reserved for individuals who have not responded adequately to at least two other DMT.

#### 4.4.3. B-Cell-Depleting Therapies: Rituximab, Ocrelizumab, and Ofatumumab

In the past decade, the traditional view of MS as predominantly T-cell mediated has shifted to the view that MS relapses involve interactions among different immune cell types, including B cells. The role of B cells in MS pathophysiology was discussed earlier. The importance of B cells is further supported by pathological studies showing that B cell infiltrates are prominent in MS, especially in the early stages and in active lesions [7,115]. As a result, B-cell-depleting therapy has been employed in MS.

CD20 is a transmembrane non-glycosylated phosphoprotein that is expressed on the surface of cells in a lineage from pre-B cells to memory B cells. Monoclonal antibodies directed against the CD20 antigen deplete CD20+ B cells via a few mechanisms: antibody-dependent cellular cytotoxicity, complement-dependent cytotoxicity, and antibody-dependent cellular phagocytosis [116]. The administration of anti-CD20 monoclonal antibodies leads to a rapid decrease in CD20+ B cells, usually within days. The depletion can be sustained for several weeks to months depending on the characteristics and dosing regimen of the specific anti-CD20 monoclonal antibody.

Rituximab is a chimeric anti-CD20 monoclonal antibody. It was initially developed as a therapy for hematological malignancies and was later found to be useful in many autoimmune diseases, including rheumatoid arthritis. Although not FDA-approved, rituximab has been used off-label in MS due to its potential efficacy, especially before the newer anti-CD20 therapies became available.

The efficacy of rituximab in MS has been demonstrated in several clinical trials. HERMES, a phase II randomized double-blind placebo-controlled study published in 2008, showed that a single course of intravenous rituximab reduced inflammatory brain lesions on MRI and clinical relapses for 48 weeks in patients with RRMS [29]. The efficacy of rituximab in primary progressive multiple sclerosis (PPMS) was also evaluated in a 96-week phase-II/III randomized placebo-controlled trial. However, between rituximab and a placebo, the proportion of patients developing confirmed disability progression, the primary endpoint of the study, did not reach significance [117]. Several other retrospective studies have shown that rituximab can reduce relapse rates and stabilize disability in both relapsing and progressive MS [118]. 

Ocrelizumab is a humanized monoclonal anti-CD20 antibody that binds with high affinity to the large extracellular loop of CD20 and induces B cell depletion via similar mechanisms to rituximab [119]. It was approved by the FDA for the treatment of active RRMS and PPMS in 2017. The standard regimen is 600 mg IV every 6 months.

Two phase III multicenter, randomized, double-blind, active-controlled trials (OPERA I and OPERA II) were performed to investigate the efficacy and safety of ocrelizumab, compared with subcutaneous interferon beta-1a, in patients with relapsing multiple sclerosis. Ocrelizumab reduced the annualized relapse rate by 46% and 47% compared interferon beta-1a. It also reduced 12-week confirmed disability progression by 40% and the number of enhancing lesions on MRI by more than 90% [30]. The clinical benefits of ocrelizumab treatment were also demonstrated in patients with PPMS in a phase III randomized, placebo-controlled trial (ORTARIO) which evaluated 732 PPMS patients for at least 120 weeks. Ocrelizumab was shown to reduce 12-week and 24-week confirmed disability progression [120]. It was also associated with less worsening on a timed 25-foot walk test, a decreased T2 lesion volume on MRI, and a lower rate of brain atrophy in patients with PPMS compared to a placebo [120]. Of note, ocrelizumab is the only FDA-approved disease-modifying therapy for PPMS.

Ofatumumab is a fully human monoclonal anti-CD20 antibody that binds to a small-loop epitope of CD20 close to the cell surface and induces B-cell depletion via both complement-dependent cytotoxicity and antibody-dependent cellular cytotoxicity [121]. It has been approved by the FDA for the treatment of RRMS and active SPMS since 2020. Ofatumumab is given monthly as a 20 mg subcutaneous injection.

In the MIRROR study, a phase II randomized, placebo-controlled trial, ofatumumab was shown to reduce the cumulative number of new enhancing lesions by 65% at week 12 compared with a placebo [121]. Two recent phase III randomized controlled trials (ASCLEPIOS I and II) in patients with RRMS compared the efficacy and safety of ofatumumab with 14 mg of oral teriflunomide daily. The ofatumumab groups showed 51% and 59% lower annualized relapse rates [73]. Ofatumumab was also associated with a lower percentage of disability worsening at 3 months and 6 months, lower disease activity on MRI and lower serum neurofilament light-chain levels [73].

Infusion/injection-related reactions are not uncommon in all anti-CD20 therapies. Common symptoms include fever, headache, rash, nausea, throat irritation, hypotension, and itchiness. These symptoms could be mitigated by premedication and slower infusion rates. Anti-CD20 therapies are also associated with hypogammaglobinemia, especially with prolonged treatment. In the randomized controlled trials of ocrelizumab and ofatumumab, lower immunoglobulin levels were not associated with serious infections [30,73]. Upper and lower respiratory infections and infections with herpes viruses have been associated with anti-CD20 treatments. PML has occurred rarely in people on anti-CD20 therapies. Screening for HIV, hepatitis B, hepatitis C, and tuberculosis and a quantitative serum immunoglobulin test are required before starting any anti-CD20 antibody.

Immunosuppressive drugs may influence tumor surveillance and thus potentially increase the risk of malignancies. In MS patients, rituximab was not associated with a long-term increased risk of cancer compared with the general population [122]. In the OPERA I and OPERA II clinical trials, malignancies were reported in four patients (0.7%) treated with ocrelizumab compared to two patients (0.2%) who received IFNβ1a [30]. In the ORATORIO trial, eleven patients (2.3%) treated with ocrelizumab developed malignancies, including four cases of breast cancer, whereas in the placebo group, malignancies were observed in two patients (0.8%) [120]. The occurrence of breast cancer in the group treated with ocrelizumab was consistent with what is typically expected based on epidemiological studies, and the incidence rate decreased during the extension studies [123]. Patients receiving ocrelizumab should follow standard breast cancer screening guidelines. In ASCLEPIOS I/II trials, no increase in rates of malignancies was observed in ofatumumab-treated patients [73]. 

B cell-depleting drugs are expected to interfere with the humoral response and thereby reduce the effectiveness of vaccines. Patients should complete any required vaccinations at least 4 to 6 weeks prior to treatment initiation. Live attenuated or live vaccines are not recommended during treatment and until B-cell recovery for any B-cell-depleting therapy and at least 6 months after the last administration of rituximab.

### 4.5. Use of Disease-Modifying Therapies in Multiple Sclerosis

#### 4.5.1. Escalation Strategy

The traditional escalation strategy involves the initiation of a low-efficacy DMT (typically IFNβ, GA, or TF) and then carefully monitoring patients using clinical assessments and MRI for disease activity evidenced by clinical relapse, new or worsening neurological signs/disabilities, or subclinical new or enlarging lesions on MRI. If the disease remains active after an adequate duration of therapy (typically 6 months or longer), treatment will be escalated to another DMT with higher efficacy. The advantage of this strategy is the safety of the low-efficacy DMT, avoiding the early use of a high-efficacy DMT with a risk of SAE such as PML and secondary autoimmunity [124]. 

#### 4.5.2. Early High-Efficacy Therapy (HET)

Age is an important factor for DMT efficacy, and MS patients benefit more from higher-efficacy therapy during the early stage of the disease at a younger age [125]. Recent evidence from both clinical trials and post-marketing real world data support that the early initiation of HET results in the more effective prevention of relapses, less disability progression, fewer cases of SPMS, and overall better preservation of brain volume and functions compared to escalation strategies [41,42,43]. A pivotal RCT of HET with comparators of lower efficacy showed that ofatumumab, ocrelizumab, and alemtuzumab are superior to teriflunomide (ASCLEPIOS), IFNβ1a (OPERA), and IFNβ1a (CARE-MS), respectively, in reducing the ARR, disability progression, new/enlarged MRI lesions, and BVL in patients with relapsing MS [30,110,111,126]. In addition, in an open-label extension (OLE) of these RCTs, patients switching from low-efficacy IFNβ therapy responded to HET similarly to those continuing on alemtuzumab and ocrelizumab regarding the ARR and MRI activity but had more disability and BVL than patients initiated on HET earlier. This supports the clinical benefits of early HET use rather than delayed use in relapsing MS for the optimization of short-term and long-term outcomes [126,127]. 

Real world data also suggest clinical benefits of HET over low-efficacy therapy as a first-line treatment for relapsing MS. A retrospective Swedish study of newly diagnosed RRMS patients reported that rituximab was superior to all other DMTs in terms of drug discontinuation and had better clinical efficacy (rate of clinical relapse and/or disease activity on MRI) compared to injectable DMFs and borderline significance compared to natalizumab and fingolimod [128]. A Danish study reported that treatment-naïve relapsing MS patients who received HET (FG, natalizumab, or alemtuzumab) as first DMT had a lower probability of first relapse and 6-month CDP compared to matched patients who received a low-/moderate-efficacy DMT (IFNβ, GA, TF, or DMF) at 4 years of follow-up [129]. Another European study involving 592 patients showed that MS patients initiated on HET (alemtuzumab or natalizumab) had lower EDSS scores after 5 years compared to those initiated on a low-/moderate-efficacy DMT (IFN, GA, TF, DMF, or FG) [130]. Another study of the MSBase group found that initial treatment with HET (FG, alemtuzumab, or natalizumab) was associated with a lower risk of conversion to SPMS compared to initial treatment with low-efficacy therapy (IFNβ or GA), and the conversion rate was lower with HET started within 5 years versus after 5 years [131]. A retrospective study based on the MSBase and Swedish MS registries reported that patients who received HET (rituximab, mitoxantrone, alemtuzumab, or natalizumab) within 2 years of clinical onset had less disability after 6–10 years compared to those who received HET 4–6 years after onset [132]. A large-scale Italian study involving 2702 patients revealed increasing differences in disability trajectory over up to 10 years of follow-up between those treatment-naïve patients initiated on HET (FG, natailizumab, mitoxantrone, or cladribine) within 13 months of clinical onset compared to propensity-score matched patients who received HET after 1 year or a longer duration of treatment with low-/moderate-efficacy DMT [133].

#### 4.5.3. Personalized Use of Disease-Modifying Therapies in Multiple Sclerosis

With more than 10 DMTs with differences in efficacy, adverse effect profiles, and routes of delivery available for relapsing MS patients who are heterogeneous in severity and prognosis, we would like to emphasize that the personalized use of DMTs should be practiced with careful consideration for individual patient characteristics. Shared decision making with consideration for disease severity and activity, risk factors for a poor prognosis, patients’ expectations, preferences, and comorbidities, and family planning is recommended.

We strongly recommend offering HET early to relapsing MS patients who present with moderate and severe clinical attacks and/or have high disease activity, especially with poor prognostic factors (Table 1). Patients should have an understanding of the efficacy and potential adverse effects of the HET and share in the decision making process regarding the choice of HET according to preference, expectations, comorbidities, pregnancy planning, especially for females, and possible availability and costs, which vary in different localities. Importantly, cladribine and alemtuzumab are usually used as second-line DMTs after the failure of or intolerance to one or two other DMTs because of adverse effect profiles, whereas B cell depletion therapies and natalizumab are commonly used as first-line DMTs for these patients. Once the choice is made and HET is initiated, patients should be reminded of the importance of compliance and monitored closely with regular clinical and MRI assessments for disease activity and blood tests for potential adverse effects as needed. NEDA-3, no evidence of disease activity based on the absence of clinical relapse, MRI disease activity, and disability progression, is commonly employed for assessments of breakthrough disease activity [134]. Breakthrough disease activity, either clinical relapse/disability progression or subclinical MRI disease activity, indicate a need to switch to another HET with different pharmacological mechanisms. Pulsed therapy with immune reconstitution (alemtuzumab and cladribine) [16,42,43] and continuous therapy requiring infrequent injection/infusion (rituximab, ocrelizumab, and ofatumumab) may help solve the problem of suboptimal compliance.

For patients presenting with clinically mild attacks without evidence of high disease activity or poor prognostic factors, we recommend the early initiation of low-/moderate-efficacy DMT (IFNβ, GA, TF, and fumaric acid derivatives) in view of the potentially severe adverse effects of high-efficacy DMTs, including infection [135] and malignancy [43]. Similarly, patients should understand the efficacy and potential adverse effects of the DMT and share in the decision of the choice of DMT according to their preference, expectations, and comorbidities and family planning. Once the DMT is initiated, patients should be reminded about compliance and monitored closely with regular assessments for neurological signs/disability, MRI disease activity, and potential adverse effects. If breakthrough disease activity occurs after an adequate treatment duration (6 months at least), early escalation to a DMT with higher efficacy is recommended. The choice of therapy will be based on the consideration of various factors including potential efficacy, adverse effects, patients’ preference, comorbidities, and family planning. Natalizumab has been shown to be superior to fingolimod in RRMS patients with breakthrough disease activity on a low-efficacy DMT [124,136], and rituximab and natalizumab therapy result in less disease activity compared to fingolimod for patients who switch from a low-efficacy DMT due to breakthrough disease [137].

#### 4.5.4. De-Escalation and Therapy Suspension

For MS patients on continuous DMT with no clinical relapse, MRI disease activity and disability progression, it is expected that therapy can continue. Switching DMTs with stable disease after one to two years is considered for patients on natalizumab who are seropositive for the JC virus antibody, especially with a high antibody index, to avoid PML. Switching from natalizumab to rituximab has been reported to be associated with a lower risk of disease reactivation compared to de-escalation to fingolimod [138]. There are few data to guide the optimal duration of treatment with DMT and whether DMT should be stopped. An observational study showed that MS patients who stopped injectable DMT after being relapse-free for ≥5 years had similar risks of relapse but higher risks of disability progression compared to those who did not stop [139]. Older adults with relapsing MS may be considered for stopping DMT [140]. It remains unclear whether MS patients on DMT still benefit from the treatment after years of clinical and radiological stability [41].

#### 4.5.5. Pregnancy, Lactation and DMT for Multiple Sclerosis Patients

In general, like people without MS, MS patients should have family and offspring; they should not be discouraged from pregnancy [41,59]. The ARR is reduced during the third trimester of pregnancy but increased/rebounds in the early postpartum period (first 4–8 weeks) [141]. The seminal pregnancy in MS (PRIMS) study observed that relapse activity diminished during pregnancy, especially during the third trimester, but increased postpartum [142]. A recent metanalysis demonstrated that pre-pregnancy, DMT use was associated with a reduction in the ARR from 0.57 before pregnancy to 0.36 during the first trimester, 0.29 during the second trimester, and 0.16 during the third trimester, with postpartum rebound to 0.85 [143]. Importantly, patients should have a well-controlled disease before pregnancy. IFNβ and GA are the only approved DMTs for use during pregnancy. Most of DMTs should be stopped before conception and during pregnancy, particularly teriflunomide, which requires rapid elimination by cholestyramine or activated charcoal (long half-life), and fingolimod, which requires a washout period of 2 months before pregnancy. Patients on fingolimod are usually recommended to switch to ocrelizumab or rituximab before stopping contraception to avoid the higher risk of relapse during pregnancy reported at 8–16 weeks post fingolimod or natalizumab discontinuation [144,145,146]. 

If a patient has persistent active disease, pregnancy should be delayed until the disease is stabilized. Pulsed therapy with alemtuzumab and cladribine followed by immune reconstitution and a disease-free state may be an option for pregnancy planning, and patients can become pregnant 4 to 6 months after their last dose of alemtuzumab and cladribine, respectively. B-cell-depleting monoclonal antibodies may be a good alternative for pregnancy planning as they have low teratogenic risk, are not associated with rebound disease activity after discontinuation, and maintain protective effects for months after an infusion. In particular, as IgG1 cannot cross the placenta in the first trimester, it confers no risk of transient B-cell depletion for infants during a first trimester exposure [147].

Breastfeeding may be associated with a lower risk of postpartum relapse, according to one metanalysis [148]. Breastfeeding is not recommended while receiving DMT except for the injectable therapies as many DMTs are present in breast milk and potentially harmful to infants [149]. 

## Figures and Tables

**Table 1:** Factors associated with a poor prognosis for disability progression in multiple sclerosis.

**Poor prognosis factors** Onset after age 50; Male; Non-white ethnicity; Smoking; Obesity (especially childhood and teenage); First attack with motor weakness; Multifocal onset; Cognitive impairment; Frequent relapses in the first 2–5 years after clinical onset; Heavy T2 lesion load (high number and volume of T2 lesions); Infratentorial lesions on MRI; Spinal cord lesions on MRI; New T2 lesion formation in the first 5 years; Presence of CSF OCBs; High level of neurofilament light subunit.

**Table 2:** Characteristics of approved and commonly used disease modifying therapies for relapsing multiple sclerosis.

Efficacy	DMT	* Reduction of ARR	Key Pharmacological Mechanisms	Important Adverse Effects	Risk of Malignancy on Long-Term Use	Safety for Pregnancy
**Low**	IFNβ	32–35% (compared to placebo)	Reduce antigen presentation and T cell proliferation, shift Th1 to Th2 response, restore suppressor function	Deranged LFT, flu-like Sx, skin reaction, depression	Nil	Safe (non-teratogenic)
**Low**	Glatiramer acetate	29% (compared to placebo)	Alter T cell differentiation to induce proliferation of anti-inflammatory lymphocytes	Skin injection site reaction; lipoatrophy	Nil	Safe
**Low**	Teriflunomide	34% (compared to placebo)	Inhibit proliferation of autoreactive B and T lymphocytes	Nausea, diarrhea, hair loss, deranged LFT, infection	Nil	Contraindi-cated in pregnancy, need accelerated elimination and organ screening USG if accidental pregnancy
**Moderate**	Dimethyl fumarate	51% (compared to placebo)	Affect Nrf2 pathway activity, reduce release of inflammatory cytokines and activate antioxidant pathways (neuroprotective effects)	Flushing, gastrointestinal symptoms, lymphopenia, infection, low risk of PML (1 in 50,000); hypertension	Nil	Uncertain, inadequate data for conclusion
**Moderate to high**	**S1P receptor modulators**					
	Fingolimod,	54% (compared to placebo)	SIP receptor modulators: induce degradation of S1P receptors, trapping of lymphocytes in secondary lymphoid tissues	Headache, bradycardia, heart block, lymphopenia, infection especially herpes virus, PML (1 in 12,000), macular oedema, liver function derangement	Increased risk of malignancy (skin basal and Merkel cell carcinoma, melanoma)	Unsafe, increased risk of CA with exposure in first trimester, washout period of 2 months before pregnancy recommended
	Siponimod	55% (compared to placebo)	similar to fingolimod	Headache, bradycardia, heart block, lymphopenia, hypertension, liver function derangement	Uncertain	Uncertain, risk of CA with exposure in first trimester unknown, likely similar to fingolimod, washout period of 10 days before pregnancy recommended
	Ozanimod	48% (compared to IFNβ1a)	similar to fingolomod	URTI, UTI, liver function derangement, bradycardia, heart block, lymphopenia, hypertension, orthostatic hypotension, back pain	Uncertain	Uncertain
**Moderate to high**	Cladribine	58% (compared to placebo)	Nucleoside analogue, induce apoptosis of lymphocytes, followed by repopulation of lymphocytes	Lymphopenia, infection (no case of PML reported)	Possible increased risk of malignancy	Uncertain, conception at least 6 months after last dose recommended
**High**	Natalizumab	69% (compared to placebo)	Bind to endothelial VCAM1 to prevent migration of lymphocytes to CNS	Infusion reaction, anti-drug antibody, infection, PML	Nil	Uncertain, SA and CA likely not elevated for exposure in first trimester
**High**	Alemtuzumab	52% (compared to IFNβ1a)	Depletion of lymphocytes, monocytes, NK cells followed by repopulation of lymphocytes	Infusion reaction, infections especially herpes virus (not PML), secondary autoimmunity, stroke, arterial dissection, hemophagocytosis	Possible increased risk of malignancy including melanoma, thyroid cancer	Uncertain conception at least 4 months after last dose
**High**	**Anti-CD 20 monoclonal antibodies**					
	Ocrelizumab	46% (compared to IFNβ1a)	Depletion of CD20+ B cells	Infusion reaction, infection, lymphopenia, hypogammaglobulinaemia	Possible increased risk of malignancy	Uncertain, low teratogenic risk, conception 2 months after last dose
	Rituximab	50% (compared to placebo)	Depletion of CD20+ B cells	Infusion reaction, infection, lymphopenia, hypogammaglobulinaemia	Nil	Uncertain, reduced B cell count in newborns, conception 1-3 months after last dose recommended
	Ofatumumab	59% (compared to teriflunomide)	Depletion of CD20+ B cells	Infection, lymphopenia, hypogammaglobulinaemia	Nil	Uncertain, conception 2 months after last dose recommended

* Reduction of annualized relapse rate according to the published clinical trials which vary in the comparators including placebo and other comparator DMT such as interferon-beta and teriflunomide. CA = congenital anomalies; SA = spontaneous abortion.

**Table 3:** Continuous and pulsed disease-modifying therapies for relapsing multiple sclerosis.

**Disease-modifying therapies for relapsing multiple sclerosis.**
*Continuous therapy*: Interferon-beta (IFNβ); Glatiramer acetate (GA); Teriflunomide (TF); Dimethyl fumarate (DMF); Sphingosine-1-phosphate receptor modulators (Fingolimod (FG), Siponimod (SP), and Ozanimod (OZ)); Natalizumab (Nat); B cell depletion therapy (rituximab, ocrelizumab, ofatumumab). *Pulsed therapy:* Alemtuzumab; Cladribine.
